# Urban–suburb disparities in pre-hospital emergency medical resources and response time among patients with out-of-hospital cardiac arrest: A mixed-method cross-sectional study

**DOI:** 10.3389/fpubh.2023.1121779

**Published:** 2023-02-20

**Authors:** Yinzi Jin, Hui Chen, Hongxia Ge, Siwen Li, Jinjun Zhang, Qingbian Ma

**Affiliations:** ^1^Department of Global Health, School of Public Health, Peking University, Beijing, China; ^2^Institute for Global Health and Development, Peking University, Beijing, China; ^3^Network Management and Quality Control Department, Beijing Emergency Medical Center, Beijing, China; ^4^Emergency Department, Peking University Third Hospital, Beijing, China; ^5^Beijing Emergency Medicine Research Institute, Beijing Emergency Medical Center, Beijing, China

**Keywords:** out-of-hospital cardiac arrest, response time, urban–suburb disparities, pre-hospital emergency medical system, health resources

## Abstract

**Aim:**

To investigate (1) the association between pre-hospital emergency medical resources and pre-hospital emergency medical system (EMS) response time among patients with Out-of-hospital cardiac arrest (OHCA); (2) whether the association differs between urban and suburbs.

**Methods:**

Densities of ambulances and physicians were independent variables, respectively. Pre-hospital emergency medical system response time was dependent variable. Multivariate linear regression was used to investigate the roles of ambulance density and physician density in pre-hospital EMS response time. Qualitative data were collected and analyzed to explore reasons for the disparities in pre-hospital resources between urban areas and suburbs.

**Results:**

Ambulance density and physician density were both negatively associated with call to ambulance dispatch time, with odds ratios (ORs) 0.98 (95% confidence interval [CI] 0.96–0.99; *P* = 0.001) and 0.97 (95% CI; 0.93–0.99; *P* < 0.001), respectively. ORs of ambulance density and physician density in association with total response time were 0.99 (95% CI: 0.97–0.99; *P* = 0.013) and 0.90 (95% CI: 0.86–0.99; *P* = 0.048). The effect of ambulance density on call to ambulance dispatch time in urban areas was 14% smaller than that in suburb areas and that on total response time in urban areas was 3% smaller than the effect in suburbs. Similar effects were identified for physician density on urban–suburb disparities in call to ambulance dispatch time and total response time. The main reasons summarized from stakeholders for a lack of physicians and ambulances in suburbs included low income, poor personal incentive mechanisms, and inequality in financial distribution of the healthcare system.

**Conclusion:**

Improving pre-hospital emergency medical resources allocation can reduce system delay and narrow urban-suburb disparity in EMS response time for OHCA patients.

## Introduction

Out-of-hospital cardiac arrest (OHCA) is a global health problem that largely contributes to mortality worldwide (1). Each year, an estimated 395,000 OHCA cases are diagnosed in the United States (2), with an overall mortality rate of approximately 90% (3). In China, OHCA tends to be growing among overall population, including urban and non-urban residents (4). As a life-threatening condition requiring urgent intervention, immediate activation of emergency medical system (EMS) response is key to saving OHCA patients' lives and better prognosis (5, 6). As the first line of EMS, pre-hospital emergency systems provide first aid on site and transfer patients to hospitals, which plays an important role in improving health outcomes (7, 8). Most studies concerning the relationship between pre-hospital systems and health outcomes of critical diseases have focused on the first aid measures taken on site (9, 10), but studies on the association between pre-hospital emergency medical resource allocation and pre-hospital EMS response time were scarce.

With the rapid development of the social economy and the growth of aging population in China, EMS has gradually emerged as an important part of healthcare systems. Patients can access EMS services by calling “120” whole 24 h, and the nearest ambulances will be dispatched according to the patient's location. Physicians, nurses, and drivers are assigned in an ambulance beforehand to provide life support to patients (11). In order to improve the quality of healthcare services, a healthcare reform with the goal of equal and guaranteed essential healthcare services for all by 2020 was launched by the Chinese government in 2019 (12, 13). Although the government has implemented a series of policies on developing EMS as part of China's healthcare system reform, regional unbalanced distribution of emergency medical resources still remains a considerable problem (14).

In China, the unbalanced distribution of EMS between urban areas and suburbs has been leading to fragmented, poorly coordinated healthcare. Patients have to migrate to urban areas to get better EMS service, which may cause medical crowding in cities as well as aggravate urban-suburb economic development gap (15, 16). Apart from that, partially owing to unequal access to pre-hospital emergency medical resources, a very pronounced gap exists in the treatments and outcomes of patients between suburb and urban areas of China (17). Evidence reveals that regional differences in emergency medical resources can result in variations in EMS response time and survival for patients with cardiovascular diseases (18). Therefore, focusing on urban–suburb disparities in pre-hospital emergency medical resources and pre-hospital EMS response time for patients with OHCA are essential.

To fill this gap, we examined the association between pre-hospital emergency medical resources and pre-hospital EMS response time among patients with OHCA; and then investigated whether the association differs between those living in urban and suburb areas. The findings can provide evidence to expand pre-hospital emergency medical resources and reduce existing urban–suburb disparities in pre-hospital EMS response time for patients with OHCA in China.

## Methods

### Quantitative data collection and study participants

The data were obtained from regular reports on the allocation of emergency medical resources at emergency medical stations, a database of patients with OHCA in the EMS, and the Beijing Statistical Yearbook (19). Regular data reports of emergency medical stations comprise the number and characteristics of physicians and ambulances at 435 emergency medical stations in 16 districts (6 urban and 10 rural areas) of Beijing in 2020. Appendix 1 shows the questionnaire for reporting data. The database contains clinical data on patients with OHCA which were reported in real time by emergency medical centers of Beijing in 2020. The Beijing 2021 Statistical Yearbook that covers January to December 2020 provides information on the area, population density, and gross domestic product (GDP) for each district of Beijing.

We merged the three data sources according to district and emergency station coding, and excluded patients who lacked EMS response time records. A unified reporting platform with a standardized format and data elements is used to report data for each emergency medical station (Appendix 2). The emergency medical center was responsible for the quality of data. A full-time data manager returns irregular or logically improper values to each emergency medical station for rechecking. Finally, a total of 435 emergency medical station directors, 838 ambulances, and 3,538 patients with OHCA were included in this analysis.

### Quantitative data measures

#### Independent variables

An ambulance was always staffed by one physician in China, which could provide life support to critically ill patients (20). Thus, the amounts of ambulances and physicians were used as indicators of the pre-hospital medical resources. We used the number of physicians per square kilometer (km^2^) to measure the quantity of pre-hospital emergency medical care professionals, and the number of ambulances per square kilometer (km^2^) t to measure the quantity of pre-hospital emergency medical equipment.

#### Dependent variables

We analyzed the following two pre-hospital EMS response times as dependent variables: (1) call to dispatch time, defined as the interval from emergency medical station operators receiving a call to dispatch an ambulance; (2) total response time, defined as the interval from emergency medical station operators receiving a call to ambulance arrival on the scene.

#### Covariates

Demographic and district covariates included patient's age (< 40, 40–69, ≥70 years), sex (male or female), location of cardiac arrest (home = 1, public = 0), season of cardiac arrest (month 1–3 = 1, 4–6 = 2, 7–9 = 3, and month 10–12 = 4), population density, area, and GDP. Urban or suburb patient was defined as the onset region recorded in the EMS.

### Qualitative data collection and measures

We used a semi-structured interview to explore the reasons for a lack of quantity and quality in emergency medical resources at emergency medical stations. Guideline for interviews was shown in Appendix 3. Interviews were conducted by two well-trained interviewers, and participation was entirely voluntary. We selected one director from each emergency medical station, so a total of 435 interviewees were enrolled. Interview questions involved ambulance dispatch management, reasons for a lack of physicians and ambulances, recruitment requirements, and staff incentives. Interviews were conducted privately in a separate room with notetaking and audio recording, which took 30 min, and were ended once data saturation was reached.

Themes were identified in advance by content analysis. The coding framework was divided into institutional- and healthcare system-level factors that influence physician retention and ambulance allocation. Each of these two levels was further coded by workload, incentives, financial support, and other specific motivating factors. NVivo 12.0 was used to analyse consumption to develop theoretical coding. Two well-trained interviewers prepared verbatim transcripts and coded the audio records. Transcripts were reviewed and discussed to revise and improve codes.

### Statistical analysis

We described the characteristics of patients with OHCA and emergency medical resources among the full sample as a whole and by groups of urban and suburb residents. Continuous and categorical variables were analyzed using the *t*-test and chi-square test (*P* < 0.05), respectively. Multivariate linear regression was performed to investigate the roles of ambulance density and physician density in pre-hospital EMS response time. Model 1 regressed each outcome of ambulance density/physician density and urban patients. Model 2 added an additional regressor of the interaction term between ambulance density or physician density and urban patients. Both models were adjusted for covariates with *P* ≤ 0.05 in the descriptive analysis. District fixed effects and patients' characteristics were added in both models to adjust for unobserved district-level and patient factors. The sign of the interaction term in Model 2 could be interpreted as whether ambulance density or physician density modified the urban–suburb disparity of pre-hospital emergency medical response time. All statistical analyses were performed using Stata SE (StataCorp., College Station, TX, USA).

## Results

### Characteristics of study participants

**Figure 1:**
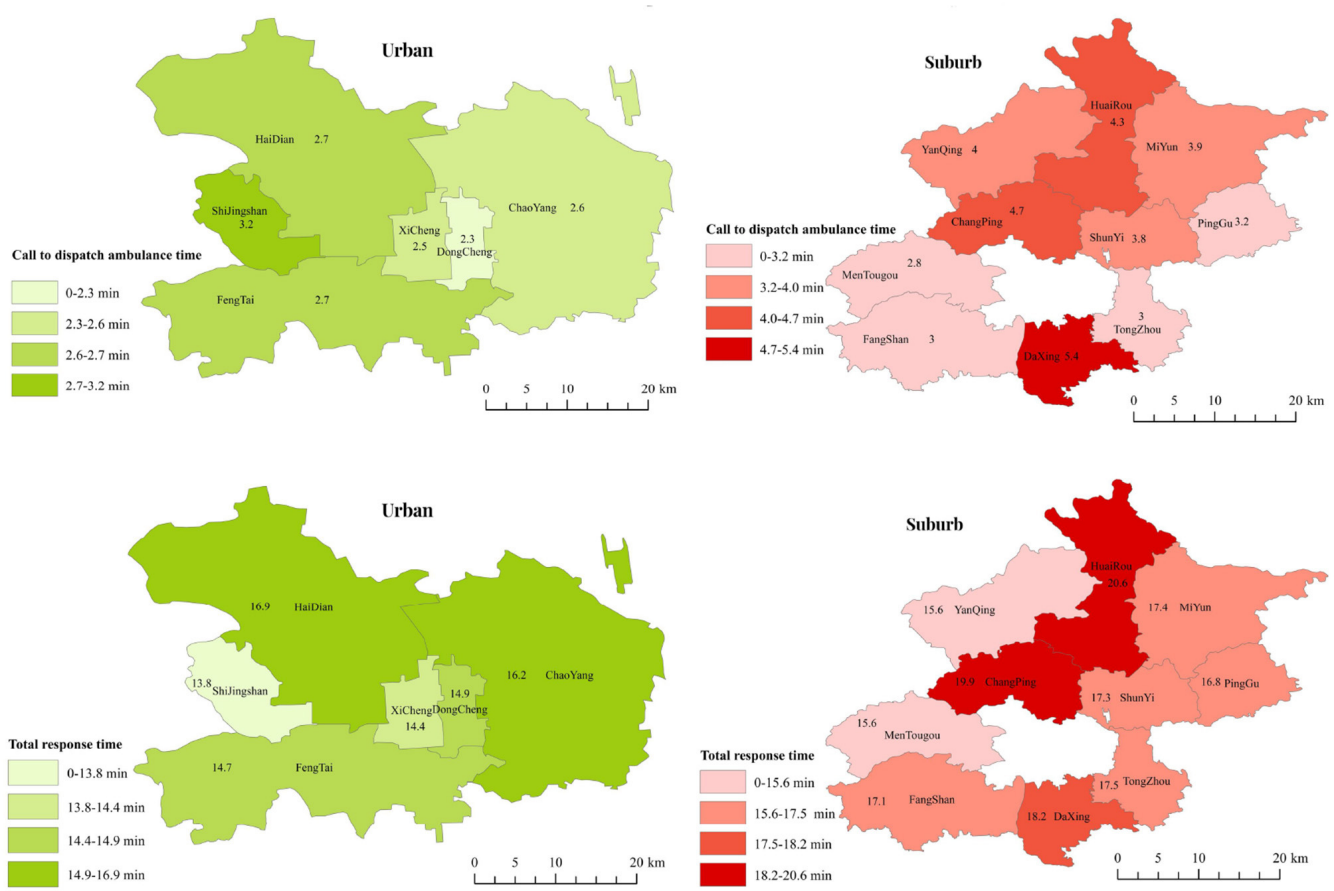
Pre-hospital emergency medical system response time for patients with out-of-hospital cardiac arrest in urban and rural areas.

This cross-sectional study enrolled 3,538 patients with OHCA, including 2,209 urban and 1,329 suburb residents. Pre-hospital EMS response time for call to ambulance dispatch was 3.08 (standard deviation [SD] 2.10) min, and total response time was 16.41 (SD 8.73) min (Figure 1). Compared with suburb patients, urban patients were older (64.93% aged ≥70 years for urban vs. 50.81% for suburb residents; *P* < 0.001), were more men (59.44 vs. 65.99%; *P* < 0.001). OHCA was most often seen between April to June in urban areas (32.35%), while it was from October to December in suburbs (36.03%). In terms of emergency medical resource allocation, urban areas had higher ambulance density (0.31 per km^2^ in urban vs. 0.03 per km^2^ in suburb areas; *P* < 0.001) and higher physician density (1.61 per km^2^ in urban vs. 0.27 per km^2^ in suburb areas; *P* < 0.001) (Table 1).

**Table 1:** Characteristics of study participants.

**Characteristics**	**Overall**	**Urban**	**Rural**	***P-value***
**Cardiac arrest patients characteristics**				
**N**	3,538	2,209	1,329	
Age, yrs, *n* (%)				< 0.001
< 40	165 (5.79)	90 (5.11)	75 (6.89)	
40–69	988 (34.67)	528 (29.97)	460 (42.28)	
≥70	1,697 (59.54)	1,144 (64.93)	553 (50.81)	
Gender, *n* (%)				< 0.001
Male	2,190 (61.90)	1,313 (59.44)	877 (65.99)	
Female	1,348 (38.10)	896 (40.56)	452 (34.01)	
Location of cardiac arrest, *n* (%)				0.442
Home	3,408 (96.33)	2,132 (96.51)	1,276 (96.01)	
Public area	130 (3.67)	77 (3.49)	53 (3.99)	
Season of cardiac arrest, *n* (%)				< 0.001
1^st^-3^th^	630 (22.11)	511 (29.00)	119 (10.94)	
4^th^-6^th^	787 (27.61)	570 (32.35)	217 (19.94)	
7^th^-9^th^	825 (28.95)	465 (26.39)	360 (33.09)	
10^th^-12^th^	608 (21.33)	216 (2.26)	392 (36.03)	
Response time, min (mean, SD)				
Call to dispatch time	3.08 (2.10)	2.62 (1.46)	3.85 (2.70)	< 0.001
Total response time	16.41 (8.73)	15.57 (7.83)	17.80 (9.89)	< 0.001
**Emergency medical system characteristics**				
Ambulance density (number per km^2^)		0.31	0.03	< 0.001
Physicians density (numbers per km^2^)		1.61	0.27	< 0.001

### Association between ambulance density and pre-hospital EMS response time

**Figure 2:**
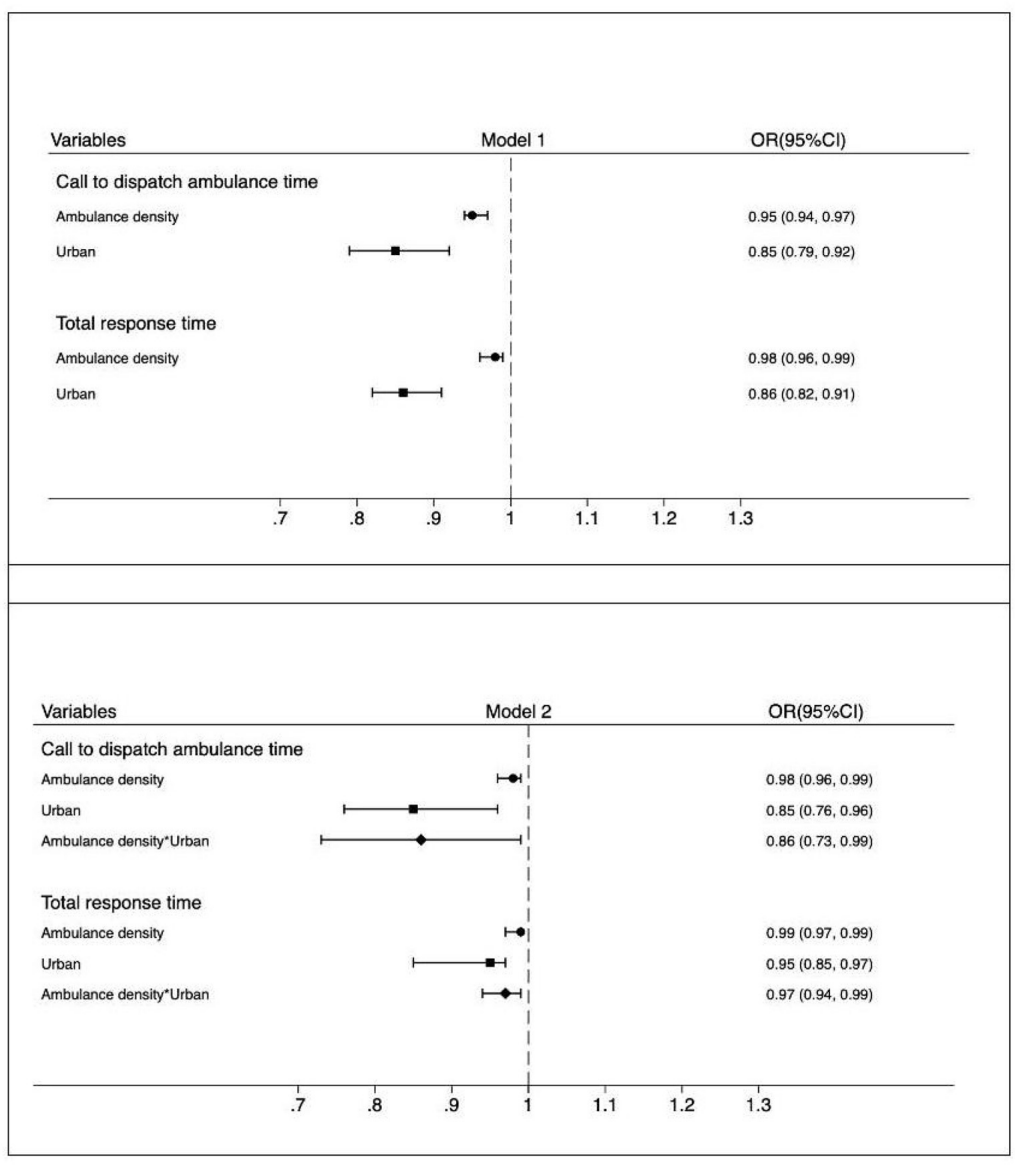
Multivariate regression for relationship between ambulance density and urban patients with out-of-hospital cardiac arrest and pre-hospital emergency medical system response time.

Figure 2 shows the odds ratio (OR) of multivariate linear regression for ambulance density and urban residence to pre-hospital EMS response time. Without adding the interaction term between ambulance density and urban residence (Model 1), ambulance density was negatively associated with call to ambulance dispatch time (OR 0.95; 95% confidence interval [CI] 0.94–0.97; *P* < 0.001) and total response time (OR 0.98; 95% CI 0.96–0.99; *P* = 0.013). Of note, we found significant disparities in call to ambulance dispatch time (OR 0.85; 95% CI 0.79–0.92; *P* < 0.001) and in total response time (OR 0.86; 95% CI 0.82–0.91; *P* < 0.001) between urban areas and suburbs. After adding the interaction term (Model 2), the negative associations between ambulance density and pre-hospital EMS response time were still significant with a larger coefficient size in both outcomes: call to ambulance dispatch time (OR 0.98; 95% CI 0.96–0.99; *P* = 0.001) and total response time (OR 0.99; 95% CI 0.97–0.99; *P* = 0.013). In terms of urban–suburb disparities, the coefficient size remained same for call to ambulance dispatch time (OR 0.85; 95% CI 0.76–0.96; *P* = 0.008), but it became smaller for total response time (OR 0.95; 95% CI 0.85–0.97; *P* = 0.015).

### Association between physician density and pre-hospital EMS response time

**Figure 3:**
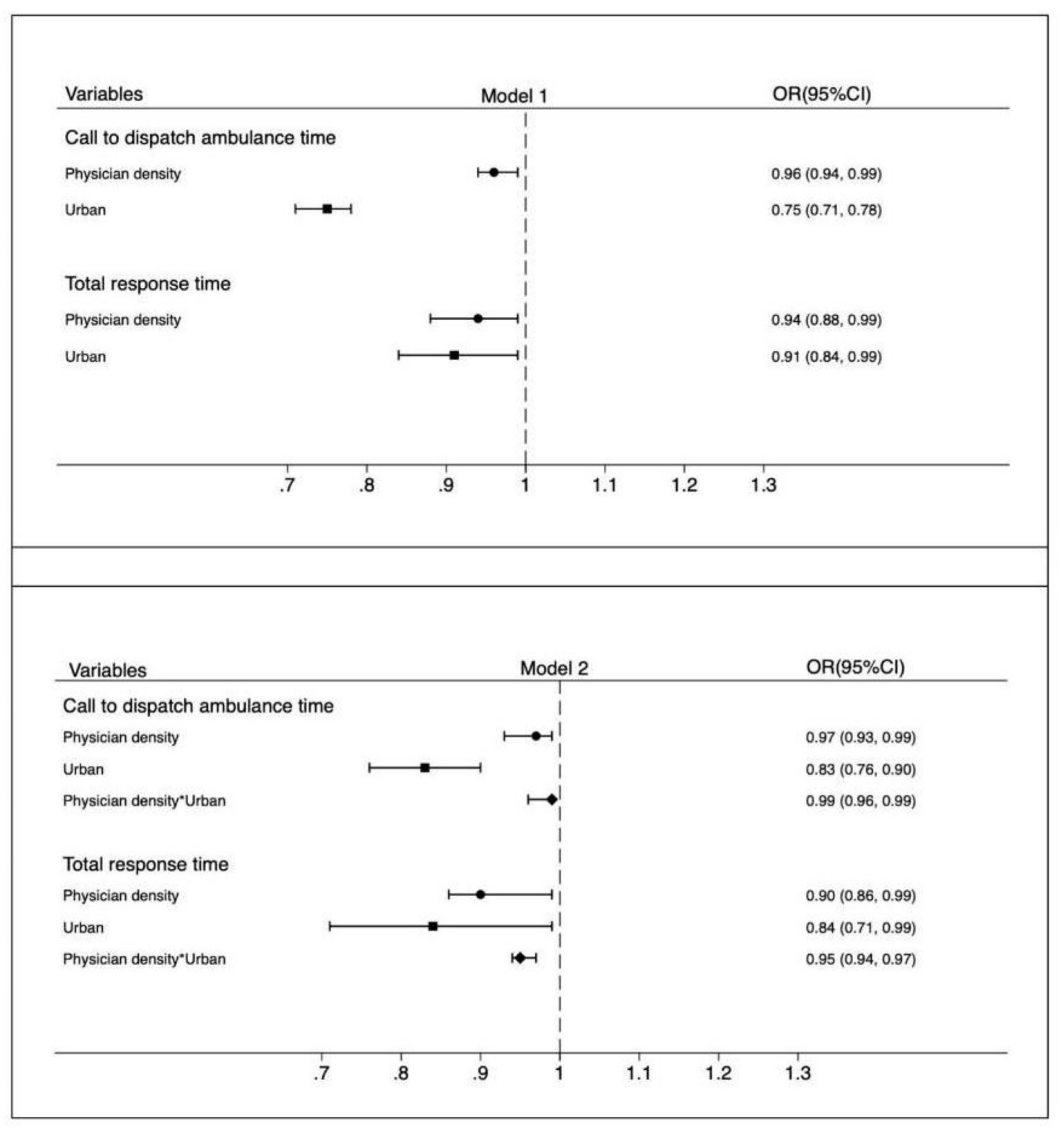
Multivariate regression for relationship between physician density and urban patients with out-of-hospital cardiac arrest and pre-hospital emergency medical system response time.

Figure 3 presents the ORs of multivariate linear regression for physician density and urban patients to pre-hospital EMS response time. Without adding the interaction term between physician density and urban patients (Model 1), physician density had a significantly negative effect on call to ambulance dispatch time (OR 0.96; 95% CI 0.94–0.99; *P* = 0.001) and on total response time (OR 0.94; 95% CI 0.88–0.99; *P* = 0.042). There were significant urban–suburb disparities in call to ambulance dispatch time (OR 0.75; 95% CI 0.71–0.78; *P* = 0.001) and in total response time (OR 0.91; 95% CI 0.84–0.99; *P* < 0.001).

After adding the interaction term (Model 2), physician density was still significantly associated with both outcomes with a larger coefficient size for call to ambulance dispatch time (OR 0.97; 95% CI 0.93–0.99; *P* < 0.001), but with a smaller coefficient size for total response time (OR 0.90; 95% CI 0.86–0.99; *P* = 0.048). Regarding urban–suburb disparities, the coefficient size was larger for call to ambulance dispatch time (OR 0.83; 95% CI 0.76–0.90; *P* < 0.001), but it became smaller for total response time (OR 0.84; 95% CI 0.71–0.99; *P* = 0.040).

### Association between pre-hospital emergency medical resources and urban–suburb disparities in pre-hospital EMS response time

The ORs of the interaction term were < 1 for the outcomes, indicating that emergency medical resources can decrease the urban–suburb disparity in Pre-hospital EMS response time for patients with OHCA and vice versa. Figures 2, 3 provide details of the interaction terms. Notably, the OR of the interaction term between ambulance density and urban residence for call to ambulance dispatch time was 0.86 (95% CI 0.73–0.99; *P* = 0.003), suggesting that the effect of ambulance density on call to ambulance dispatch time in urban patients was 14% smaller than that in suburb patients. The effect of ambulance density on total response time in urban patients was also smaller than that in suburb patients (interaction term OR 0.97, 95% CI 0.94–0.99; *P* = 0.045). Similar patterns were observed when considering the interaction term between physician density and urban residence for call to ambulance dispatch time (OR 0.99; 95% CI 0.96–0.99; *P* = 0.019) and for total response time (OR 0.95; 95% CI 0.94–0.97; *P* < 0.001).

### Reasons for lack of quantity and quality among physicians and ambulances in suburb areas

In consistency with the results of the quantitative analysis, the interview shows that suburb areas had lower density of physicians and ambulances than urban areas. According to the themes that were pre-identified using an advanced coding framework, financial factors and incentive mechanisms were the main reasons for physician resignation. Directors of emergency medical stations indicated that physicians who worked in suburb areas had more complaints about lower income and welfare than their counterparts in urban areas. Inequality in the financial distribution between suburb and urban emergency medical stations affected the number and categories of ambulances purchase, leading to a lack of quantity and quality in ambulance allocation in suburb areas. It was noticed that emergency medical centers had limited funding, thus they could not afford enough high-quality ambulances to meet all needs of emergency medical stations.

## Discussion

There is a large disparity in the mortality rate of cardiovascular diseases between urban and suburb areas in China (21, 22). In this study, we investigated the impact of pre-hospital emergency medical resources on *p*re-hospital EMS response time for patients with OHCA. Furthermore, we examined whether the pre-hospital emergency medical resources have a moderating effect on the existing urban–suburb gap in pre-hospital EMS response time.

In this study, several key findings are highlighted. There was a negative independent association of ambulance density with pre-hospital EMS response time among patients with OHCA, which is in line with previous studies. Chocron et al. found that high ambulance density was associated with shorter response time and better health outcome of patients with OHCA (23). Holmén et al. showed that shorter ambulance response time decreased mortality in OHCA (24). Another study enrolling 26,479 OHCA patients showed that ambulance shortage caused less ambulance dispatch within 1 km when regional ambulance services demand increased, resulting in a response delay in OHCA scene (25). Therefore, increasing access to ambulance services might be beneficial to improve patient's outcomes with OHCA, especially in the context of large regional population growth.

There was an inverse association between physician density and pre-hospital EMS response time among OHCA patients. Pre-hospital physicians play an important role in the emergency medical care (26). An investigation of patients with ST-elevation myocardial infarction in China found that high-quality and sufficient pre-hospital physicians can contribute to shorter emergency response time and better patient's clinical outcomes (27). A study of 25,580 patients with OHCA conducted in Japan showed that an increase in pre-hospital physicians per 100,000 individuals was likely to reduce the delay in emergency medical service activation (28). Demands for emergency medical services are huge in China owing to its large and aging population. Low income and heavy workloads give rise to a shortage of pre-hospital physicians in Beijing, a city of China (29). Improving pre-hospital work mechanisms, enriching personal incentive policies, and increasing welfare are potential measures to raise the quality of emergency medical services.

We found that pre-hospital emergency medical resources were associated with a disparity in pre-hospital EMS response time among patients with OHCA between urban and suburb areas. An existing gap in EMS response time for OHCA between urban and suburb areas was also found in many previous studies (17, 30). Our findings revealed that urban areas had higher densities of ambulances and physicians than suburb areas. Increasing pre-hospital emergency medical resources could narrow the urban–suburb gap in response time for OHCA, consistent with similar studies (31, 32). A well-documented contributor to urban–suburb disparities in EMS response time is the shortage and unequal distribution of resources, especially for regions with large square of areas (33–35). Although exploring the mechanisms of the association between pre-hospital emergency medical resources and increased urban–suburb disparities in OHCA response time was beyond the scope of this study, our findings can help to narrow the urban–suburb disparity in the allocation of emergency medical resources.

Our qualitative analysis revealed the reasons for the lack of physicians and ambulances in suburb areas. Financial factors commonly were the main drivers of the shortage of physicians in suburb areas. Low income and inappropriate personal incentive mechanisms lead to poor job satisfaction among physicians in suburbs, which is consistent with previous studies. Li et al. showed that salary was positively associated with the retention rate of physicians in rural and underserved areas (36). Furthermore, suburb physicians have less opportunities to work in a private sector (37). Additionally, personal incentive mechanisms of pre-hospital physicians are defective, because physician's performance is only evaluated according to the number of ambulances dispatch, not the treatment. Therefore, physicians may prefer working in urban, well-developed areas because of higher income. Changing the incentive mechanism is critical to increasing personal income, which is essential to improve the shortage and work motivation of pre-hospital physicians.

Another finding of our qualitative analysis was that the number of ambulances allocated in suburb areas was lower than that in urban ones. The disparity in funding distribution in the healthcare system is a main factor that affects the shortage and quality of ambulances in suburbs. The Chinese government has injected massive funding into the healthcare system, yet disparities in the funding between EMS and other sectors of the healthcare system remain (13, 20). Therefore, our findings provide evidence that pre-hospital development requires greater attentions from local governments because it may indirectly influence the activation of EMS.

## Limitations

First, this was an observational study, which limits the ability to draw any causal inference from our findings. The results should not be interpreted as describing the effect of emergency medical resources on increasing gaps between urban and non-urban areas in pre-hospital emergency medical service response time for OHCA. Second, this study was conducted in one city only selecting patients with onset in 2020, which during the COVID-19 pandemic period. Thus, the results may lack representativeness owing to selection bias and pandemic affect. Third, this study only focused on response time related to pre-hospital emergency medical system rather than outcomes or treatment variables of OHCA patients. Lastly, physician-led ambulance was present in most cities of China, then the effect of paramedic-led ambulance on response time for OHCA and the model of physician-staffing ambulances were not assessed in this study.

## Conclusion

Given the continuous increase in OHCA and the large population of China, enhancing pre-hospital emergency medical resources reserve may be a potential measure to narrow the urban-suburb gaps in EMS response time and reduce system delay for OHCA patients, and indirectly improve clinical outcomes of the patients.

## Data availability statement

The original contributions presented in the study are included in the article/Supplementary material, further inquiries can be directed to the corresponding author.

## Ethics statement

This project was approved by the Peking University Health Science Center Institutional Review Board (IRB00001052-21020). Informed consent was obtained from all participants prior to questionnaire administration.

## Author contributions

Conceptualization: QM and YJ. Data curation and writing—original draft: YJ, HC, and HG. Formal analysis, investigation, and methodology: YJ, HC, HG, SL, and JZ. Funding acquisition and supervision: QM. Validation: SL and JZ. Writing—review and editing: QM, SL, and JZ. All authors contributed to the article and approved the submitted version.
