# Supplementary material for: Urban–suburb disparities in pre-hospital emergency medical resources and response time among patients with out-of-hospital cardiac arrest: A mixed-method cross-sectional study
Source: Front Public Health. 2023 Feb 20;11:1121779. doi: 10.3389/fpubh.2023.1121779 (PMC9986292; doi:10.3389/fpubh.2023.1121779)
Supplement: Supplementary file 1 [file Data_Sheet_1.docx]

Appendix 1:

Questionnaire for regular report data of the Beijing Center for Prehospital Care

Name of Emergency Medical Stations:

Address of Institution: Beijing City

District/County

Street/Township

Name: Phone Number:

Date: / /

**Questionnaire Purpose and Confidentiality Statement**

This questionnaire was jointly developed by the Beijing Center for Prehospital Care and the School of Public Health, Peking University. We will strictly keep the survey subjects confidential, and the data collected will be used only for academic research. All the information will not appear in any publication, nor will it be used for any commercial purpose or administrative evaluation. In order to ensure the scientificity and effectiveness of the survey, please fill in truthfully. Thank you for your cooperation!

1. The service scope of this site includes: _____ streets and _____ communities; the service coverage of permanent population includes: _____ people (the permanent population refers to the population who has lived in the community for six months or more)
2. The service coverage radius of this site is: _____
3. Within 5 kilometers; ② 5-10 kilometers; ③ more than 10 kilometers
4. The total staff of this site: _____

Among them: Doctors: _____, Number of men: _____

Among them: Nurses: _____, Number of men: _____

Other personnel (drivers and stretchers, etc.): _____ , number of men: _____

1. Among the doctors on this site:

Graduate: _____

Undergraduate: _____

Junior College: _____

Technical Secondary: _____

High School: _____

1. Among the nurses on this site:

Graduate: _____

Undergraduate: _____

Junior College: _____

Technical Secondary: _____

High School: _____

1. Among the titles of doctors on this site:

Senior Level: _____

Vice Senior Level: _____

Intermediate Level: _____

Teacher/Assistant Level: _____

Primary Level: _____

1. Among the titles of nurses on this site:

Senior Level: _____

Vice Senior Level: _____

Intermediate Level: _____

Teacher/Assistant Level: _____

Primary Level: _____

1. Among the doctors on this site:

Under 25: _____

25～34 years old: _____

35～44 years old: _____

45 years old and above: _____

1. Among the doctors on this site:

Under 25: _____

25～34 years old: _____

35～44 years old: _____

1. years old and above: _____
2. There are _____ ambulances on this site; the number of ambulances equipped with electrocardiograph and simple ventilator is _____
3. Considering the number of people served by this site, do you think the medical staff on this site can meet the demand?
4. Yes; ② No; ③ Not sure
5. Considering the population served by this site, do you think the ambulances equipped on this site can meet the demand?
6. Yes; ② No; ③ Not sure

Appendix 2:

The registry data elements

| Category | Data Elements |
| --- | --- |
| Patient demographics | Age, sex, race, marriage, education, employment, insurance status |
| Medical history and risk factors | Height, weight, hypertension, hyperglycemia, hyperlipidemia, current smoker, family history of heart disease, prior cardiac history, prior revascularization |
| Prehospital treatment | Location of onset, onset date/time, transfer status, arrival date/time, date/time of first medical contact, date/time of first ECG, bypass ED/CCU |
| Presenting features and evaluation | ECG findings, consciousness, heart rate, systolic/diastolic blood pressure, cardiogenic shock, heart failure, Killip class, troponin concentration, serum creatinine, mini-GRACE risk score, preliminary diagnosis |
| In-hospital medication | Antiplatelet agents (aspirin, Clopidogrel/tegrillo), anticoagulant agents (warfarin, unfractionated heparin, low molecular weight heparin, bivalirudin, fondaparinux), intensive statin, β-blockers |
| In-hospital reperfusion strategy | LVEF assessment, Time to sign informed consent, primary PCI date/time (Cath lab activation date/time, Cath lab arrival date/time, door-to-balloon time), thrombolytic date/time, rescue PCI date/time, CABG date/time, recanalization date/time, TIMI class |
| In-hospital outcomes | Heart failure, length of stay, total cost, clinic outcomes (discharge/death/transfer to other hospitals) |
| Hospital Discharge | Principal discharge diagnosis, discharge medicines (DAPT, ACEI/ARB, statin, β-blockers), discharge counseling (Smoking cessation counseling, weight control counseling, blood pressure lowering medications, lipid lowering medications, glucose lowering medications, antithrombotic, follow up scheduling) |

ECG, Electrocardiograph; ED, emergency department; CCU, coronary care unit; PCI, percutaneous coronary intervention; LVEF, left ventricular ejection fraction; CABG, coronary artery bypass graft; DAPT, dual antiplatelet therapy; ACEI, angiotensin-converting enzyme inhibitor; ARB, angiotensin receptor blocker.

Appendix 3:

**Interview Guide Director**

Name of interviewee： Gender： Occupation：

Institution： Date： / /

Brief description of the interview environment process：

Investigator： Recorder：

1. What is your management of emergency medical dispatch like?
2. Do you think you have any difficulties in managing staff?
3. What is personnel incentive mechanism in your emergency medical station, as well as the results or problems of these practices, and how to improve them?
4. How do you feel about the motivation and motivation of your employees? If their work is positive, what motivates them? If they are passive, what are the factors that cause them? How can it be improved? Do you think there are any ideas, ideas or practices to mobilize the enthusiasm of the staff?
5. What measures have you taken to attract and retain talent? What are the problems and how can they be improved?
6. What measures have you taken to encourage employees to take part in training or academic education? What are the problems or difficulties in personnel training? How can it be improved?
7. What do you think is the reason for the lack of healthcare professionals?

Table S1: The director of emergency medical centre perceived effects of work mechanism on pre-hospital emergency medical resources

| **Themes** | **Quotes** |
| --- | --- |
| Management of emergency medical dispatch | “ We follow the principle of proximity. Our dispatcher will dispatch nearest ambulances according to patients location.”  “ For critically ill patients, we give a priory to dispatching ambulances.”  “ Ambulances will be allocated with physicians to provide some life support for patients on the site and transferring process.” |
| Personnel incentive mechanism | “ Pre-hospital physicians will be gave subsidized according to the numbers of ambulance dispatching they involved.” |
| Attract and retain talent | “ Increasing salary and provide better welfare, such as housing allowance and performance allowance. ”  “ Give them opportunities to rise up their careers.” |
| Reason for the lack of pre-hospital emergency medical resources? | “ The work mechanism for pre-hospital physicians is day-night shift, which may influence the normal life for them, especially for female physicians have children. ”  “ Compared to their colleagues in urban area, the salary and welfare of pre-hospital physicians may be not as good as them.”  “The limited funding cannot afford sufficient quantity of ambulances due to high price.” |
